# The productivity illusion: performance measurement and the invisibility of digital clinical work

**DOI:** 10.1016/j.fhj.2026.100546

**Published:** 2026-06-24

**Authors:** Jehan Al Fannah

**Affiliations:** Royal Hospital, Ministry of Health, Muscat, Oman

**Keywords:** Value-based healthcare, Productivity, Digital health, Performance measurement, Artificial intelligence, Teleconsultation, Workforce

## Abstract

Clinical value is now produced in two distinct registers: one that requires physical presence, and one that does not. Performance measurement frameworks, however, were built for mainly the first. Where digital work is counted, it is rarely counted equitably; where it is counted equitably, it is rarely connected to the outcomes it produces. This structural gap, which is termed here value-time-place misalignment, gives rise to the productivity illusion: a system that appears busy, measurable and accountable, while systematically failing to capture the outcomes that it purports to deliver. Drawing on teleconsultation, artificial intelligence (AI)-supported decision-making, remote multidisciplinary teams and digital follow-up, this paper demonstrates how current metrics penalise efficiency, suppress innovation and render care increasingly illegitimate to the patients it serves. Redefining the unit of healthcare work is not a technical adjustment; it is a foundational one.

## Introduction

*A specialist reviews a deteriorating patient’s monitoring data at 7am from home and adjusts the care plan before the morning handover. The admission is avoided. The productivity framework records nothing.*

Healthcare systems are no longer bound by place or time. Nurses monitor patients remotely between shifts. Pharmacists review medication alerts from home. Physicians join multidisciplinary teams (MDTs) across geographies, outside scheduled hours. Patients manage chronic conditions through apps, report symptoms asynchronously, and access clinical advice without leaving their homes. The systems designed to measure, fund and govern healthcare, however, still assume that work happens in a room, at a scheduled time, with a patient physically present. Where digital work is counted, it is rarely counted equitably. Where it is counted equitably, it is rarely connected to the outcomes it produces.

The NHS illustrates this tension with clarity. Productivity continues to be tracked through finished consultant episodes, referral-to-treatment times and outpatient attendance volumes, metrics that drive commissioning decisions, inspection frameworks and workforce planning. Against this backdrop, NHS England’s expansion of virtual wards represents precisely the category of clinical work that existing frameworks cannot adequately capture.^1^ Each of the NHS 10 Year Plan’s three structural shifts, from hospital to community, analogue to digital, and treatment to prevention, depends on resolving this measurement gap as a precondition for delivery.^2^

This paper introduces the productivity illusion in healthcare: the systemic conflation of presence with productivity, activity with effectiveness, and location with work. Prior scholarship has examined critiques of activity-based funding, value-based care frameworks and telehealth reimbursement policy as distinct problems; what has not been named is the common structural source from which each derives.

## An outdated theory of work in a digital age

Performance measurement in healthcare was built for a specific model of work: clinician in clinic, patient in chair, interaction countable, time billable. This is not a neutral administrative choice; it is a foundational one, a set of prior assumptions about what work is that decides what gets counted before anyone starts counting. It defines work as presence, value as activity, and productivity as throughput. It is, in its structure, an industrial model applied to a professional service.

Institutional visibility constitutes institutional existence. Foucault observed that the clinic made the patient’s body legible by fixing it in a defined space and time; the same logic now governs clinical labour.^3^ The clinician who works within designated spaces and scheduled hours is seen, counted and credited; the one who works outside them, asynchronously, remotely, across geographies, is not. The most adaptive clinical work, the work that prevents harm, avoids admissions and crosses institutional boundaries, is precisely the work least legible to performance frameworks designed to count what occurs inside designated clinical spaces. This gap is particularly consequential for the NHS 10 Year Plan’s shifts from treatment to prevention and hospital to community, both of which generate clinical value in precisely the spaces and times that volume-based proxies cannot see.^2^

The World Health Organization (WHO) Health Systems Performance Assessment Framework acknowledges that productivity in healthcare must ultimately be measured against health outcomes, responsiveness and financial protection, not input activity.^4^ Operational performance management in most health systems, however, remains anchored to volume-based proxies: appointments, procedures, contacts and bed utilisation. The gap between what frameworks aspire to measure and what organisations count is where the productivity illusion lives.

Digital transformation has exposed this theory of work by violating it. The COVID-19 pandemic compressed a decade of digital adoption into 18 months.^5^ Teleconsultation, remote MDTs, AI-supported decision tools and asynchronous digital follow-up became routine. What changed was not merely the delivery channel; it was the spatial and temporal structure of clinical work itself.

Here, this structural gap is termed value-time-place misalignment: the failure of performance systems to account for clinical value produced outside designated clinical spaces and standard working hours. Porter’s foundational argument makes the case directly: value in healthcare is outcomes achieved per unit cost, not outputs generated per unit time or place.^6^ Donabedian established decades ago that activity measures belong to the process domain and cannot substitute for outcome accountability, but process proxies have proved far more administratively durable than the outcome measures that they were meant to approximate. What has not previously been named is the distinct structural mechanism, value-time-place misalignment, through which digital transformation has exposed and compounded this longstanding failure.

## The illusion in practice

Four domains make the misalignment concrete.

Teleconsultation. Telephone and video consultations are now routine in the NHS and many comparable health systems, a structural change accelerated by the COVID-19 pandemic and sustained by patient and clinician preference.^5^ Where measurement has not kept pace, however, the productivity signal remains distorted. Reimbursement structures continue to weight teleconsultation below in-person contact, or exclude it from activity data entirely in some settings. Following Australia’s permanent extension of telehealth under the Medicare Benefits Schedule in December 2021, activity-based funding models failed to credit remote consultations at equivalent value to face-to-face care, constraining adoption among providers operating under volume-dependent revenue structures.^7^ The adoption challenge has been substantially resolved; the measurement challenge has not.

AI-supported clinical decisions. Machine learning tools for imaging analysis, sepsis prediction, medication safety and risk stratification are demonstrating clinical utility across multiple domains.^8^ Their integration creates a paradox: if an AI tool reduces the time that a clinician takes to reach a safe decision, standard frameworks record less clinician time and, therefore, less productivity. Efficiency appears as a performance deficit. The NHS Topol Review (2019) identified this dynamic explicitly, concluding that AI adoption at scale would require fundamental revision of job planning, workforce modelling and productivity frameworks, frameworks that, more than 5 years on, remain substantially unchanged.^9^

Remote MDTs. Multidisciplinary meetings conducted remotely enable participation from clinicians geographically excluded from in-person equivalents, broadening the representativeness of collective decision-making. A respiratory consultant participating in a daily virtual ward review via video conference performs work that appears in no job plan and no productivity return. Such participation, conducted between commitments without a dedicated room or scheduled slot, is absorbed as invisible overhead, generating coordination value that no performance report acknowledges. When unacknowledged work of this kind accumulates across a clinician’s working day, it constitutes a hidden burden on individual workload and wellbeing that performance frameworks not only fail to measure, but actively render invisible.

Digital follow-up. Asynchronous digital follow-up, including app-based symptom monitoring, patient-reported outcomes and automated titration protocols, has demonstrated effectiveness in managing long-term conditions and supporting earlier detection of deterioration.^10^ It removes outpatient contacts from the denominator while improving outcomes, a combination that current frameworks read as reduced activity, without connecting the mechanism to the result.

Across all four domains, the pattern is consistent: work has changed; measurement has not. Each example shares a common structural cause, not a failure of data collection, but a failure of definition. The framework that follows names that cause.

## The productivity illusion: a conceptual framework

The productivity illusion rests on four conflations that performance measurement systems have failed to interrogate.

Busy ≠ effective. High activity volume is not equivalent to high clinical impact. A ward round reviewing 20 patients in 2 hours may be less effective than one reviewing 12 with deliberation and structured handover. The OECD health systems analysis makes clear that productivity, properly defined, requires adjustment for quality and outcomes, not merely activity counts.^11^

Present ≠ productive. Physical attendance is not a proxy for value creation. A clinician reviewing remote monitoring data from home generates clinical value. A clinician present on a ward but awaiting a scan result, a pharmacy release or a bed manager decision consumes time without generating proportionate value. Presence-based measurement cannot distinguish between these states.

Measured ≠ meaningful. The metrics health systems find easiest to collect, attendances, waiting times, bed days, procedures, are not the metrics that best capture performance. They are proxies selected for administrative convenience, sustained by path dependency: organisations have built reporting infrastructure, regulatory accountability and funding flows around them, creating institutional inertia that incremental reform cannot displace.

Location ≠ working. This is the conflation which the previous three depend on, and the one least examined in the literature. Performance systems assume that work occurs in a designated clinical space during designated hours. Digital healthcare has made this assumption visible by breaking it. A nurse documenting care plan updates at 11pm, a physician reviewing postoperative telemetry alerts from home: real clinical value appears in the patient record, but in no workload model. A system that cannot see this work will systematically undercount the most adaptive practice in the organisation.

These four conflations carry direct patient consequences. A system that measures presence rewards the clinician who sees the patient face to face over the one who prevents the deterioration remotely. It incentivises the appointment over the outcome, the attendance over the recovery. For a generation of patients who manage banking, education and social connection without attending a physical location, this model is increasingly illegitimate. Large-scale NHS data show that working-age digital natives already demonstrate the highest rates of patient portal adoption in NHS secondary care.^12^ This is not a generational preference to be managed; it is a baseline that will only intensify as younger cohorts, currently constrained by structural access barriers, age into autonomous healthcare use. The demographic shift is already under way.

## Policy implications for health systems

Resolving the productivity illusion requires structural reform across five interconnected domains.

The NHS 10 Year Plan (2025) commits to three key structural shifts: from hospital to community, from analogue to digital, and from treatment to prevention.^2^ Each shift depends, as a precondition, on resolving the measurement gap that this paper describes. A shift from hospital to community requires that clinical value produced in community and home settings is counted and credited equivalently to hospital-based work. A shift from analogue to digital requires that performance frameworks capture digital clinical work as productive activity. A shift from treatment to prevention requires outcome-based measurement that credits avoided admissions, early detection and remote deterioration management. Without measurement reform, each of these shifts risks being adopted structurally but undermined operationally, as the systems through which clinical work is recognised and rewarded continue to privilege the physical and the countable over the digital and the effective.

Redefine the unit of measurement from attendance to contribution. Digital contacts, asynchronous clinical decisions and remote MDT participation must be incorporated into standard workload models and job planning frameworks across all healthcare systems. In the NHS, this means revising the programmed activities (PAs) framework to formally recognise clinical work performed outside scheduled sessions and designated locations, work that currently sits beyond job plan visibility entirely.

Reform reimbursement to reflect outcomes achieved. Commissioning frameworks should credit hospital admissions avoided through virtual ward care and remote monitoring, not solely contacts delivered.^13^ The NHS Long Term Workforce Plan (2023) commits to expanded digital integration, but does not address the productivity frameworks governing how digitally enabled work is counted or funded; this omission must be corrected in forthcoming operational planning cycles.^14^ Australia’s permanent extension of telehealth under the Medicare Benefits Schedule demonstrates that activity-based funding frameworks can be structurally updated to incorporate remote care delivery at national scale; despite its imperfect initial value equivalence, the pathway it created provides a replicable template.^7^

Account for distributed workforce activity. Digital work recording systems must capture clinical contributions made outside designated spaces and standard hours, making the full scope of clinical labour visible to planners and commissioners.

Align AI adoption with productivity credit. Time saved through AI-assisted decision-making must register as efficiency gain rather than reduced throughput, removing a structural disincentive to adoption that current frameworks embed by design. In practice, this requires updating clinical coding conventions to capture AI-assisted care pathways as distinct, creditable activity, rather than recording faster, safer care as an absence of billable work.^8^

Commission cross-system evaluation of existing digital measurement pilots to identify scalable models for place-agnostic, time-agnostic performance accounting. This challenge is not confined to high-income, anglophone systems. GCC health systems, including Oman, the UAE and Saudi Arabia, have made substantial documented investments in telemedicine infrastructure and national digital health platforms,^15^ yet have not published equivalent reform of the activity-based performance frameworks governing how that digital work is measured and funded. Singapore’s model of outcome-sensitive resource allocation, linking subsidy structures to patient-level outcomes across its integrated financing framework, offers partial comparative precedent for accountability beyond activity counting.^16^ These contexts illustrate that value-time-place misalignment is neither geographically nor economically bounded; its resolution will require cross-system learning that existing evaluation infrastructure is not yet configured to support.

Two further barriers to implementation warrant acknowledgement for balance. Commissioning risk aversion presents a structural challenge: organisations operating under financial pressure favour proven, auditable activity metrics over outcome-based alternatives that carry greater attribution uncertainty, slowing reform even where policy intent is clear. Measurement attribution complexity compounds this: demonstrating that a remotely delivered intervention produced a specific patient outcome is methodologically more demanding than counting an attendance, and requires investment in evaluation capability that has not been systematically funded. Both barriers reinforce the case for structured cross-system pilot evaluation rather than immediate system-wide reform.

## Conclusion

Healthcare management inherited a theory of work designed for an industrial era: presence counted, time billed, output measured at the door. Digital transformation has made that theory increasingly inadequate. Clinical value is now produced across geographies, across time zones, and in ways that many measurement systems were never designed to capture.

The productivity illusion, defined as the systematic confusion of location, presence and activity with value, is not a measurement lag. It is a structural commitment to a theory of work that no longer fully describes how healthcare is delivered, or how patients, particularly the generations now entering the system as its primary users, expect to receive it.

Health systems that fail to redefine productivity for the digital age risk a compounding deficit: rewarding the wrong work, exhausting the right clinicians, and building institutional distance from the patients and professionals they depend upon. Measurement reform is not peripheral to the digital transition; it is its precondition. The measurement system is not a neutral observer. It is the most powerful signal that a health system sends about what it values. That signal must reflect where healthcare is, not where it once was.

## CRediT authorship contribution statement

**Jehan Al Fannah:** Writing – review & editing, Writing – original draft, Conceptualization.

## Ethics approval and consent to participate

Not applicable. This article does not involve human participants, human data, human tissue, or animal experiments and therefore did not require ethics committee approval or informed consent.

## Funding

This research received no specific grant from any funding agency in the public, commercial, or not-for-profit sectors.

## Declaration of generative AI and AI-assisted technologies in the writing process

During the preparation of this work, the author used Claude Sonnet 4.6 (Anthropic) to assist with drafting, structural refinement and editorial development. After using this tool, the author reviewed and edited the content and takes full responsibility for the content of the published article. This disclosure is made in accordance with the journal’s policy on generative AI and AI-assisted technologies in the writing process.

## Declaration of Competing Interest

The authors declare that they have no known competing financial interests or personal relationships that could have appeared to influence the work reported in this paper.
